# Predicting Liver Stiffness Approximately 12 Months Ahead Using Serial Transient Elastography

**DOI:** 10.1155/cjgh/1261185

**Published:** 2026-10-27

**Authors:** Ming Lei, Shoukang Zou, Qing Liu, Yangling Li, Jing Han, Yungang Zhang, Chunwen Pu

**Affiliations:** ^1^ Department of Hemodialysis Dalian Public Health Clinical Center No. 269, Gui Bai Road, Lu Gang, Ganjingzi District Dalian Liaoning China; ^2^ Unit 4-Department of Geriatric Medicine The Fourth People’s Hospital of Chengdu No.8 Huli-West First-Alley, Jinniu District Chengdu Sichuan China; ^3^ Department of Laboratory Medicine Liaoning Electric Power Central Hospital. No. 2, Wanghu Road, Heping District Shenyang Liaoning China; ^4^ Department of Gastroenterology West China Hospital of Sichuan University No. 37, Guoxue Lane, Wuhou District Chengdu Sichuan China wchscu.cn; ^5^ Biobank for Biological Samples and Data Dalian Public Health Clinical Center No. 269, Gui Bai Road, Lu Gang, Ganjingzi District Dalian Liaoning China

**Keywords:** liver stiffness measurement, longitudinal prediction, machine learning, temporal validation, transient elastography

## Abstract

**Background:**

Liver stiffness measurement (LSM) is widely used to assess chronic liver disease, but a single measurement does not capture longitudinal change. We evaluated whether serial LSMs and routine clinical indicators could predict continuous LSM approximately 12 months later.

**Methods:**

This retrospective longitudinal study included patients with at least two historical LSMs and a follow‐up LSM 270–450 days after the index date. The LSM closest to 365 days was the outcome, and each patient contributed the earliest eligible task. Patients were separated chronologically into a train set (index date through 2022), test set (2023), and validation set (2024). Nested expanding temporal cross‐validation selected the model, which was then fixed and compared with longitudinal benchmarks. Patient‐level bootstrapping provided 95% confidence intervals (CIs).

**Results:**

The analysis included 715 patients (train, 434; test, 178; validation, 103). Random forest was selected. In the test set, its mean absolute error (MAE) was 3.622 kPa (95% CI, 2.828–4.769), *R* ^2^ was 0.456 (0.240–0.550), and calibration slope was 1.043 (0.612–1.475). In the validation set, MAE was 2.989 kPa (2.149–4.149), *R* ^2^ was 0.432 (−0.141–0.575), and calibration slope was 0.951 (0.513–1.598). In the test set, the model reduced MAE by 8.443 kPa (4.833–12.605) versus linear‐trend extrapolation; however, the last observed LSM performed better, with a paired last‐observed‐LSM‐minus‐model MAE difference of −0.658 kPa (−1.168 to −0.187). Historical LSM summaries were the dominant features.

**Conclusions:**

The locked model showed reproducible out‐of‐time performance and near‐ideal calibration point estimates but did not outperform the last observed LSM. Independent multicenter validation is required before clinical translation.

**Trial Registration:** ClinicalTrials.gov identifier: NCT06123858

## 1. Introduction

Liver fibrosis, as the core pathological process driving chronic liver disease (CLD) progression, links initial hepatic injury to severe complications such as cirrhosis and hepatocellular carcinoma through mechanisms involving hepatic stellate cell activation, extracellular matrix deposition, and inflammatory cytokine‐mediated cascades [1]. While elastography‐based liver stiffness measurement (LSM) has revolutionized noninvasive fibrosis assessment through techniques like transient elastography and shear wave elastography, current reliance on single static measurements fails to capture the dynamic nature of fibrosis progression or regression [1, 2]. Dynamic LSM monitoring—encompassing longitudinal assessments interpreted alongside confounding factors like inflammation, operator variability, and patient physiology—may offer clinical value by enabling detection of changes over time and evaluation of treatment response [3–5].

Despite its promise, dynamic monitoring faces critical challenges, including the lack of standardized protocols, universally accepted cutoff values for longitudinal changes, and limited integration with other biomarkers like serum panels or microRNAs [1, 6]. Future research priorities must address these gaps through large‐scale prospective studies validating LSM trajectories against clinical endpoints, establishing consensus on measurement intervals and quality controls, and developing multimodal models that combine dynamic LSM with molecular and imaging markers.

Existing prediction models have two key research gaps [7, 8]. First, most models rely on cross‐sectional data, neglecting LSM time‐series features and individual heterogeneity in disease progression. Although longitudinal studies and registries in chronic hepatitis B and nonalcoholic steatohepatitis (NASH) have been designed to collect serial LSM and outcome data [7, 8] and time‐series LSM can reflect disease changes following alcohol abstinence or resumed alcohol use [9], machine learning algorithms suitable for time‐series data remain underutilized in this field. Second, there is a scarcity of quantitative studies systematically analyzing how historical LSM summaries, measurement timing, acquisition context, and routine clinical variables jointly influence predictive accuracy. Coupled with the absence of standardized protocols for data acquisition and uneven measurement intervals [10], this gap limits the clinical translation of prediction models.

To address these gaps, this study aims to fill the application gap of longitudinal LSM data in liver stiffness prediction. By constructing targeted machine learning models—which integrate longitudinal LSM time‐series features and multidimensional clinical indicators to capture dynamic disease patterns—this research realizes the shift from static single‐point assessment to dynamic trend prediction of continuous LSM approximately 12 months after an index date. This advancement, empowered by machine learning’s ability to capture complex temporal associations, evaluates temporal reproducibility, calibration, and incremental value against transparent longitudinal benchmarks, thereby providing a quantitative basis for subsequent multicenter and multimodal validation.

## 2. Methods

### 2.1. Study Population and Prediction Task

This retrospective longitudinal study used routine clinical follow‐up records. Each date on which a patient underwent LSM was treated as a candidate index date, and all LSMs on or before that date constituted the measurement history. The primary analysis required at least 2 valid LSMs by the index date and a follow‐up LSM 270–450 days after that date; the LSM closest to 365 days within this window was used as the continuous outcome. Only the chronologically earliest eligible prediction task was retained for each patient.

The data were divided in calendar order according to the index date of each patient’s retained task. Patients with index dates on or before December 31, 2022, entered the train set; those with index dates from January 1 through December 31, 2023, entered the test set; and those with index dates from January 1 through December 31, 2024, entered the validation set. The train set was used for algorithm comparison, hyperparameter selection, and final model fitting; the test set was used for the primary out‐of‐time performance evaluation; and the validation set was used for validation in a subsequent time period. Each patient contributed only one task, so there was no patient overlap among the three sets.

### 2.2. Transient Elastography and Predictors

Structured sex data were used to describe the cohorts in Table 1 but were not included as model predictors. Diagnosis text was used to summarize nonexclusive evidence of hepatitis B, hepatitis C, alcohol‐related liver disease, fatty liver, and autoimmune or cholestatic disease for cohort description in Supporting Table 2. These indicators were not used in model training, feature‐importance analysis, or subgroup definitions.

**TABLE 1:** Characteristics of the analysis sets and overall sex comparisons**.**

Characteristic	Unit	Train set (*n* = 434)	Test set (*n* = 178)	Validation set (*n* = 103)	Overall male (*n* = 448)	Overall female (*n* = 267)	*p* across sets	*p* male vs female
Sex	*n* (%)						0.054	Not applicable
Male		283 (65.2%)	111 (62.4%)	54 (52.4%)	448 (62.7%)			
Female		151 (34.8%)	67 (37.6%)	49 (47.6%)		267 (37.3%)		
Age at index	years	49.0 (41.0–56.0)	51.0 (43.0–59.0)	48.0 (39.5–56.0)	48.0 (41.0–56.0)	51.0 (42.5–59.0)	0.057	0.005
Index liver stiffness	kPa	6.3 (5.0–9.9)	6.7 (5.3–9.4)	6.8 (5.0–9.3)	6.8 (5.1–10.2)	6.2 (4.9–9.2)	0.439	0.065
First observed liver stiffness	kPa	7.2 (5.1–11.8)	6.5 (4.9–11.6)	6.4 (5.0–9.1)	7.2 (5.2–11.8)	6.3 (4.7–10.4)	0.465	0.008
Future liver stiffness	kPa	6.1 (4.9–8.9)	6.9 (5.3–8.9)	6.2 (5.0–9.1)	6.5 (5.1–9.2)	5.9 (4.8–8.4)	0.157	0.026
Historical LSM records	count	2.0 (2.0–2.0)	2.0 (2.0–3.0)	2.0 (2.0–3.0)	2.0 (2.0–3.0)	2.0 (2.0–3.0)	0.030	0.632
Available LSM history	days	287.0 (143.5–632.2)	345.5 (115.2–1004.8)	377.0 (140.0–1021.5)	296.0 (133.0–709.0)	329.0 (119.0–806.0)	0.092	0.866
Prediction horizon	days	358.5 (315.0–394.5)	362.0 (329.0–390.8)	349.0 (321.0–378.0)	358.5 (321.8–392.0)	355.0 (322.0–387.5)	0.143	0.262
Controlled attenuation parameter (CAP)	dB/m	228.0 (192.2–266.0)	221.5 (180.0–255.8)	234.0 (193.5–276.0)	229.0 (195.0–269.2)	223.0 (180.0–260.0)	0.169	0.023
ALT	U/L	23.2 (15.8–34.3)	22.9 (15.4–35.1)	25.6 (16.6–35.1)	26.3 (18.1–38.5)	17.8 (13.7–27.2)	0.520	< 0.001
AST	U/L	21.6 (17.2–27.7)	19.2 (15.5–28.4)	28.8 (21.2–38.1)	21.9 (17.7–29.3)	21.8 (16.6–29.6)	< 0.001	0.156
Total bilirubin	μmol/L	13.8 (10.7–18.9)	18.1 (14.8–24.7)	18.7 (14.2–23.9)	16.3 (12.5–22.2)	15.1 (10.6–19.1)	< 0.001	< 0.001
Platelet count	10^9^/L	176.0 (126.0–215.5)	176.5 (134.0–224.0)	190.0 (146.0–233.5)	175.0 (131.0–214.0)	184.5 (134.2–234.0)	0.182	0.054
Reported LSM IQR/median ratio	ratio	0.2 (0.1–0.2)	0.2 (0.1–0.2)	0.1 (0.1–0.2)	0.2 (0.1–0.2)	0.2 (0.1–0.2)	0.244	0.133

*Note:* Values are presented as *n* (%) or median (interquartile range). For *p* across sets, sex composition was compared using the Pearson chi‐square test and continuous measures using the Kruskal–Wallis H test. For *p* male vs female, all 715 patients were pooled and continuous measures were compared between males and females using the Mann–Whitney *U* test without adjustment for analysis set. All tests were two‐sided, and *P* values were not adjusted for multiple comparisons.

All LSMs in this study were obtained by transient elastography using FibroScan Touch 502 (Echosens, Paris, France) [11]. Controlled attenuation parameter (CAP) was recorded in dB/m [12], alanine aminotransferase (ALT) and aspartate aminotransferase (AST) in U/L, total bilirubin in μmol/L, and platelet count in 10^9/L. Multiple LSMs recorded for the same patient on the same day were represented by their median, and laboratory measurements were matched to the result obtained on or most recently before the index date. The LSM interquartile‐range‐to‐median ratio was also recorded to describe measurement dispersion [13, 14].

Predictors were restricted to information available on or before the index date. They included the number of historical LSMs; the most recent, first, mean, and median LSM; LSM dispersion and slope; observation duration and measurement intervals; and age, CAP, ALT, AST, total bilirubin, and the time from each corresponding measurement to the index date. Platelet count was used only in the auxiliary FIB‐4 analysis. Within each training fold, missing values were median‐imputed and accompanied by missingness indicators; all preprocessing parameters were estimated exclusively from the corresponding training data.

### 2.3. Model Development and Temporal Validation

Candidate algorithms comprised ridge regression [15], random forest [16], and histogram gradient boosting [17]. Nested expanding temporal cross‐validation was performed in the train set, jointly incorporating preprocessing, hyperparameter selection, and outer‐fold error estimation to reduce optimism from model selection [18]. The algorithm with the lowest mean outer‐fold mean absolute error (MAE) was selected as the final model, refitted on the complete train set, and then fixed before sequential application to the 2023 test set and the 2024 validation set. Neither the test set nor the validation set was used for model selection, hyperparameter tuning, preprocessing refitting, or model debugging. The 2023 test set was used for the primary final performance evaluation, and the 2024 validation set was used for validation in a subsequent time period.

### 2.4. Statistical Analysis

Sex was reported as *n* (%) and compared among the train, test, and validation sets using a two‐sided Pearson chi‐square test. Continuous measures were reported as medians (interquartile ranges) and compared across the three sets using two‐sided Kruskal–Wallis H tests. Overall sex comparisons pooled all 715 patients and used two‐sided Mann–Whitney *U* tests to compare continuous measures between males and females.

Model performance was evaluated using MAE, root mean squared error (RMSE), and the coefficient of determination (*R* ^2^). Calibration was evaluated using the intercept and slope from regressing observed values on predictions, together with mean prediction error [19]. Reference benchmarks included the last observed LSM, first observed LSM, historical mean LSM, and linear‐trend extrapolation, allowing model performance to be evaluated in the context of simple and interpretable longitudinal prediction strategies. Patient‐level bootstrap resampling with 1000 replicates was used to estimate 95% confidence intervals (CIs) for all metrics and paired MAE differences.

To evaluate the marginal contribution of individual predictors, two prespecified feature‐level analyses were conducted for the 21 features locked in the primary analysis. Leave‐one‐feature‐out (LOFO) ablation removed one feature at a time, refitted the same algorithm with the locked parameters in the train set, and calculated “MAE after feature removal − full‐model MAE”; a positive value indicated increased error after removal. Permutation importance held the full model fixed, randomly permuted one feature among patients within each evaluation cohort, and calculated “MAE after permutation − original full‐model MAE”; a positive value indicated model reliance on that feature. Each feature was permuted 100 times, and 95% CIs for both LOFO and permutation results were estimated using 1000 patient‐level bootstrap replicates. The 2023 test set and 2024 validation set were used only for out‐of‐time evaluation and not for model fitting, tuning, or feature selection. Because historical LSM summaries are correlated, permutation importance characterizes feature reliance in the locked model, whereas LOFO characterizes marginal contribution after refitting; together, they reveal information sharing and substitutability among correlated longitudinal LSM features.

Auxiliary analyses described model performance by number of historical LSMs, numerical index‐LSM range, and prediction horizon. FIB‐4 was calculated as age × AST/[platelet count × square root of ALT] [20] and evaluated only among patients with complete data within a 90‐day window. Analyses were performed using Python 3.12.4 and scikit‐learn 1.8.0 [21], and reporting was informed by the Transparent Reporting of a multivariable prediction model for Individual Prognosis Or Diagnosis plus Artificial Intelligence (TRIPOD + AI) recommendations [22]. Exploratory k‐means clustering used the silhouette coefficient to select the number of clusters and bootstrap estimates of the adjusted Rand index (ARI) to assess internal stability [23, 24].

## 3. Results

### 3.1. Characteristics of the Analysis Sets and Overall Sex Comparisons

The full dataset contained 557,881 records, including 11,863 records with an LSM value. After 1184 records without a usable date were excluded, 10,679 dated LSM records from 3983 patients remained. Same‐day consolidation yielded 9789 patient‐day visits. The 270–450‐day target rule generated 2577 candidate tasks among 972 patients. After requiring at least two historical LSM values and retaining the earliest eligible task for each patient, the primary cohort comprised 715 patients: 434 in the train set, 178 in the 2023 test set, and 103 in the 2024 validation set. No patient appeared in more than one set (Figure 1A,B; Table 1).

**FIGURE 1:**
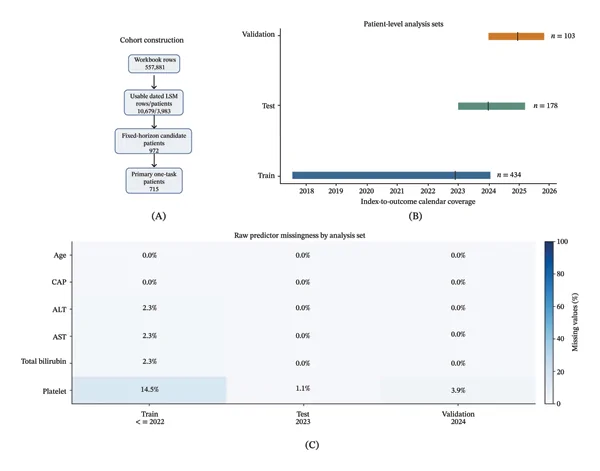
Cohort construction, temporal separation, and predictor completeness. (A) Flow diagram of primary‐cohort construction. (B) Train set (through 2022), test set (2023), and validation set (2024) defined by index date; lines represent the time from index to outcome, and there was no patient overlap among cohorts. (C) Raw proportions of missing predictor values in each cohort.

The median age in the primary cohort was 49.0 (41.0–57.0) years; 448 patients (62.7%) were male and 267 (37.3%) were female. Median index, first, and target LSMs were 6.5 (5.0–9.7), 6.8 (5.0–11.6), and 6.3 (5.0–8.9) kPa, respectively. Patients had a median of 2 (2–3) historical LSMs, a historical observation duration of 305 (133–733) days, and an actual prediction interval of 357 (322–392) days. Overall median CAP was 227 (191–266) dB/m, ALT was 23.4 (15.8–34.8) U/L, AST was 21.8 (17.2–29.4) U/L, total bilirubin was 15.8 (11.8–21.3) μmol/L, and platelet count was 179.0 (132.3–219.0) × 10^9/L.

The proportions of females in the train, test, and validation sets were 34.8%, 37.6%, and 47.6%, respectively; the overall comparison of sex composition across the three sets yielded *χ* ^2^ = 5.820, df = 2, and *p* = 0.054. The number of historical LSMs differed among the three sets (*H* = 6.984, *p* = 0.030), as did AST (*H* = 40.583, *p* < 0.001) and total bilirubin (*H* = 65.793, *p* < 0.001); all other overall comparisons of continuous measures had *p* ≥ 0.057. In the overall sex comparison pooling all 715 patients, females were older (*p* = 0.005), whereas males had higher first LSM (*p* = 0.008), future LSM (*p* = 0.026), CAP (*p* = 0.023), ALT (*p* < 0.001), and total bilirubin (*p* < 0.001). Median index LSM was 6.8 (5.1–10.2) kPa in males and 6.2 (4.9–9.2) kPa in females (*p* = 0.065). These overall sex comparisons were not adjusted for analysis set (Table 1).

Age, CAP, and the principal LSM measures were complete for all 715 patients. ALT, AST, and total bilirubin were each missing for 10 patients (1.4%), and platelet count was missing for 69 patients (9.7%); platelet missingness was 14.5%, 1.1%, and 3.9% in the train, test, and validation sets, respectively. In the 2023 test set, 148/178 patients (83.1%) had complete FIB‐4 inputs within the 90‐day lookback window. Diagnosis text was recorded for every patient, with nonexclusive evidence of hepatitis B in 658, hepatitis C in 3, alcohol‐related liver disease in 9, fatty liver in 82, and autoimmune or cholestatic disease in 11 patients (Figure 1C; Supporting Table 2).

### 3.2. Candidate‐Model Selection and Performance in the Test and Validation Sets

Using the 434‐patient train set with index years through 2022, ridge regression, histogram gradient boosting, and random forest were compared across four expanding outer temporal folds. Their mean validation MAEs (standard deviations) were 4.281 (1.286), 3.400 (0.707), and 3.000 (0.674) kPa, respectively (Figure 2A,B; Supporting Table 1). Random forest met the predefined selection rule of the lowest mean outer‐fold MAE and was therefore selected as the final model, with a maximum‐feature proportion of 0.7 and a minimum leaf size of 3; its fold‐specific training and next‐temporal‐block validation MAEs are shown in Figure 2C. The complete random‐forest pipeline was subsequently refitted using all 434 train‐set patients and fixed. The 2023 test set included 178 patients, in whom the locked model achieved an MAE of 3.622 (2.828–4.769) kPa, RMSE of 7.256 (4.933–9.779) kPa, and *R* ^2^ of 0.456 (0.240–0.550). The fixed pipeline was then applied to the 103 patients in the 2024 validation set, yielding an MAE of 2.989 (2.149–4.149) kPa, RMSE of 6.079 (3.127–9.251) kPa, and *R* ^2^ of 0.432 (−0.141–0.575) (Table 2; Figure 3A,C,D). Among the same 2023 test patients, the MAEs of the last observed LSM, first observed LSM, historical mean LSM, and linear‐trend extrapolation were 2.964 (2.303–3.791), 4.324 (3.341–5.348), 3.218 (2.551–4.012), and 12.066 (8.194–16.288) kPa, respectively. The paired difference in MAE between the last observed LSM and the model was −0.658 kPa (95% CI: ‐1.168 to −0.187) (Figure 3B). In the test set, the calibration intercept was −0.772 (−4.087 to 2.543) kPa, the calibration slope was 1.043 (0.612–1.475), and the mean prediction error was 0.356 (−0.676–1.367) kPa (Figure 3C).

**FIGURE 2:**
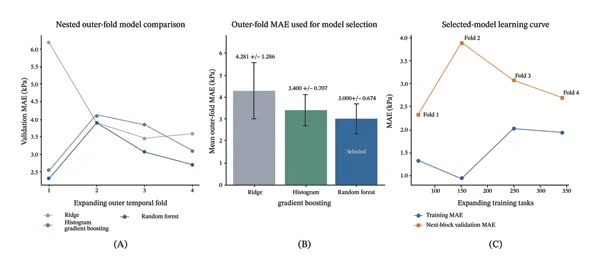
Candidate‐model performance and final selection in the train set. (A) Mean absolute error (MAE) of the three candidate models across four expanding outer temporal folds. (B) Mean and standard deviation of outer‐fold MAE for each model, on which random‐forest selection was based. (C) Random‐forest training and validation MAE in each temporal fold.

**TABLE 2:** Performance of the locked random forest and simple benchmarks in the 2023 test set**.**

Method	MAE, kPa (95% CI)	RMSE, kPa (95% CI)	*R* ^2^ (95% CI)	Baseline minus ML MAE, kPa (95% CI)
Locked random forest	3.622 (2.828–4.769)	7.256 (4.933–9.779)	0.456 (0.240–0.550)	Reference ML model
Last observed LSM	2.964 (2.303–3.791)	5.802 (3.907–7.687)	0.652 (0.468–0.752)	−0.658 (−1.168 to −0.187)
Baseline LSM	4.324 (3.341–5.348)	8.627 (6.080–10.988)	0.232 (−0.263–0.446)	0.701 (−0.010–1.340)
Historical mean LSM	3.218 (2.551–4.012)	6.247 (4.274–8.089)	0.597 (0.427–0.670)	−0.404 (−0.843 to 0.036)
Linear trend extrapolation	12.066 (8.194–16.288)	30.517 (19.604–40.143)	−8.616 (−28.897 to −2.199)	8.443 (4.833–12.605)

*Note:* Values are patient‐level bootstrap estimates with 95% confidence intervals. A positive “benchmark minus machine learning model” MAE difference indicates lower error for the random forest, whereas a negative value indicates lower error for the last observed LSM.

**FIGURE 3:**
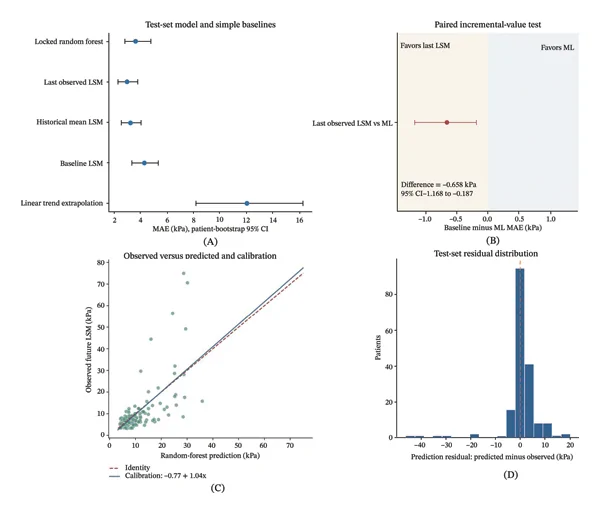
Performance, benchmark comparison, and model diagnostics in the 2023 test set. (A) MAE of the random forest and four simple benchmarks with patient‐level bootstrap 95% confidence intervals. (B) Paired benchmark‐minus‐random‐forest MAE differences; positive values indicate lower error for the random forest. (C) Observed versus predicted LSM with the calibration line. (D) Distribution of prediction residuals (predicted minus observed values).

### 3.3. Feature‐Level Ablation and Incremental Value Analysis

Starting from the locked full random forest, LOFO ablation and permutation‐importance analyses were performed for each of the 21 predictors (Figure 4). Permutation‐importance rankings were consistent across the two temporal holdout cohorts. In the 2023 test set, the MAE increases for hist_median, hist_mean, and last_lsm were 0.439 (95% CI: 0.191–0.676), 0.426 (0.151–0.708), and 0.323 (0.133–0.519) kPa, respectively; the corresponding values in the 2024 validation set were 0.616 (0.284–0.922), 0.504 (0.162–0.794), and 0.373 (0.114–0.604) kPa. hist_median and hist_mean are the median and mean of all historical LSMs through the index date and represent the patient’s prior stiffness level, whereas last_lsm is the LSM closest to the index date. The principal predictive information was concentrated in representations of longitudinal LSM history, with the remaining variables supplementing current clinical status and measurement context.

**FIGURE 4:**
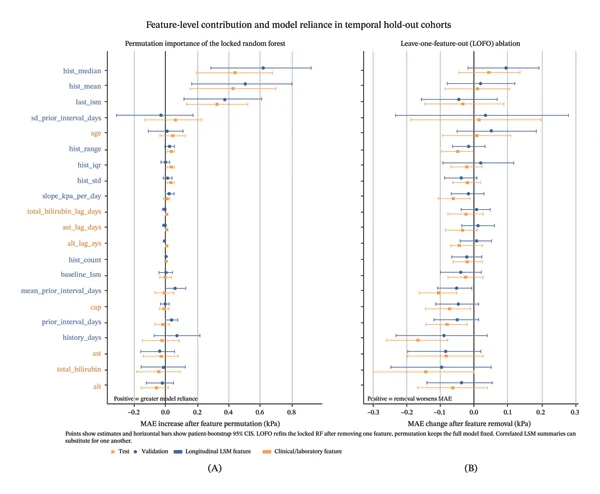
Feature‐level ablation and permutation importance. (A) Feature‐wise permutation importance for the locked random forest in the test and validation sets. (B) Leave‐one‐feature‐out (LOFO) ablation. Positive values indicate an increase in MAE after permutation or removal of a feature; horizontal lines are patient‐level bootstrap 95% CIs. Blue and orange labels denote longitudinal LSM features and clinical/laboratory features, respectively.

LOFO showed that changes in MAE after removal of a single feature were small. In the 2023 test set, removal of hist_median and age increased MAE by 0.042 kPa (95% CI: ‐0.046–0.135) and 0.008 kPa (−0.093–0.108), respectively; the corresponding increases in the 2024 validation set were 0.093 kPa (−0.018–0.191) and 0.050 kPa (−0.086–0.184). The three historical LSM summaries ranked highest in the permutation analysis in both cohorts, indicating that the model relied primarily on prior stiffness level and its historical distribution. The small changes after removal of individual features in LOFO were consistent with information sharing and compensation among correlated longitudinal summaries.

### 3.4. Auxiliary, Subgroup, and Sensitivity Analyses

In the 90‐day complete‐case auxiliary analysis, 148/178 patients (83.1%) in the 2023 test set had complete FIB‐4 inputs. The fixed random forest had an MAE of 3.651 (2.767–4.594) kPa, lower than the 4.073 (3.048–5.278) kPa achieved by the FIB‐4 linear mapping; the last observed LSM had an MAE of 2.926 (2.251–3.694) kPa in the same subset (Figure 5B). This result further contextualized the task alignment of the longitudinal multivariable framework for continuous‐LSM prediction.

**FIGURE 5:**
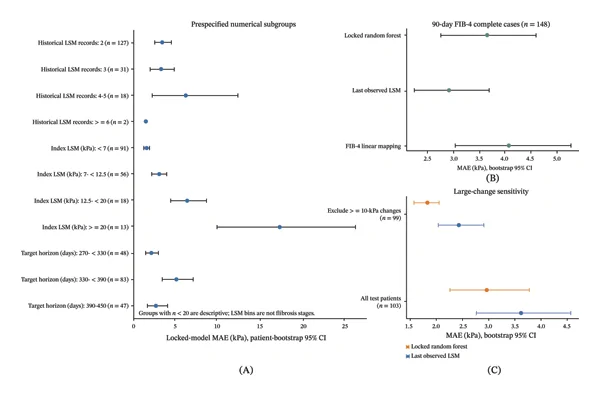
Subgroup and robustness analyses in the 2023 test set. (A) Random‐forest MAE by number of historical LSMs, index‐LSM range, and target horizon; groups with fewer than 20 patients are presented descriptively. (B) MAE of the random forest, last observed LSM, and FIB‐4 linear mapping in the 90‐day complete‐case subset. (C) MAE of the random forest and last observed LSM among all patients and after excluding those with an absolute LSM change ≥ 10 kPa. Error bars are patient‐level bootstrap 95% confidence intervals.

In the 2023 test set, 127, 31, 18, and 2 patients had 2, 3, 4–5, and ≥ 6 historical LSM records, respectively. In the repeated‐task sensitivity analysis, the ≥ 6‐record group included 5 patients and 7 tasks and had an MAE of 2.924 kPa (95% CI: 1.633–3.888). Results by index‐LSM range and target horizon are shown in Table 3 and Figure 5A. After exclusion of 12 patients with an absolute index‐to‐target LSM change ≥ 10 kPa, the direction of the primary comparison remained stable (Figure 5C).

**TABLE 3:** Subgroup performance in the 2023 test set**.**

Subgroup definition	Subgroup	Patients	Tasks	MAE, kPa (95% CI)	RMSE, kPa (95% CI)	*R* ^2^ (95% CI)	Interpretation
Historical LSM records	2	127	127	3.383 (2.479–4.467)	6.708 (4.085–9.050)	0.452 (0.137–0.556)	Reported with patient‐level bootstrap interval
Historical LSM records	3	31	31	3.272 (1.968–4.802)	5.381 (3.033–7.614)	0.584 (−6.005 to 0.740)	Reported with patient‐level bootstrap interval
Historical LSM records	4–5	18	18	6.154 (2.213–12.346)	12.374 (2.836–20.733)	0.396 (−2.721 to 0.502)	Descriptive only: fewer than 20 patients
Historical LSM records	≥ 6	2	2	1.450 (1.337–1.563)	1.454 (1.337–1.563)	−3.316 (−3.316 to 0.000)	Descriptive only: fewer than 20 patients
Index LSM range (kPa; numerical only)	< 7	91	91	1.517 (1.206–1.867)	2.213 (1.655–2.782)	−1.091 (−2.647 to −0.129)	Reported with patient‐level bootstrap interval
Index LSM range (kPa; numerical only)	7‐ < 12.5	56	56	2.988 (2.180–3.941)	4.499 (3.042–6.104)	−2.405 (−4.200 to −0.826)	Reported with patient‐level bootstrap interval
Index LSM range (kPa; numerical only)	12.5‐ < 20	18	18	6.339 (4.380–8.614)	7.936 (5.447–10.189)	−0.935 (−4.589 to −0.232)	Descriptive only: fewer than 20 patients
Index LSM range (kPa; numerical only)	≥ 20	13	13	17.328 (9.901–26.289)	22.633 (13.471–30.636)	−0.059 (−0.943 to 0.244)	Descriptive only: fewer than 20 patients
Target horizon (days)	270‐ < 330	48	48	2.093 (1.459–2.870)	3.240 (2.005–4.434)	−0.039 (−0.801 to 0.477)	Reported with patient‐level bootstrap interval
Target horizon (days)	330‐ < 390	83	83	5.076 (3.433–7.018)	9.601 (5.763–12.960)	0.459 (0.183–0.564)	Reported with patient‐level bootstrap interval
Target horizon (days)	390–450	47	47	2.617 (1.604–4.028)	5.089 (2.251–7.945)	0.385 (−0.329–0.626)	Reported with patient‐level bootstrap interval

*Note:* Random‐forest prediction performance is summarized by number of historical LSMs, index‐LSM range, and prediction horizon; subgroups with fewer than 20 patients are presented descriptively.

The highest silhouette coefficient selected *k* = 2. Across 100 bootstrap samples in the train set, the ARI had a mean of 0.933 (standard deviation 0.076), indicating high internal stability of the two trajectory groups; these groups were labeled Cluster 1 and Cluster 2 (Supporting Figure 1).

## 4. Discussion

The principal finding of this study is that a random forest based on longitudinal transient elastography and routine clinical information predicted continuous LSM approximately 12 months ahead in the patient‐separated 2023 test set and 2024 validation set. In these two later cohorts, the model achieved MAEs of 3.622 and 2.989 kPa, *R* ^2^ values of 0.456 and 0.432, and calibration slopes of 1.043 and 0.951, respectively. Relative to unconstrained linear‐trend extrapolation, the model reduced MAE by 8.443 kPa (95% CI: 4.833–12.605) in the 2023 test set, and the direction remained stable after excluding 12 patients with large changes, demonstrating the framework’s adaptability to sparse and irregular real‐world longitudinal records. The last observed LSM served as a strong transparent benchmark and provides a clear comparator for future enriched models.

The clinical value of serial LSM provides an important rationale for this prediction task. In chronic hepatitis B, longitudinal changes in transient‐elastography LSM have been associated with antiviral treatment and ALT normalization [25]. Transient‐elastography measurements in mixed CLD cohorts can predict liver‐related complications and mortality [26], and prospective data in nonalcoholic fatty liver disease have also shown that both increases and decreases in serial LSM are associated with subsequent liver‐related events [27]. Building on this evidence, our study converts repeated LSM from longitudinal description into an individual‐level continuous numerical prediction, providing a quantitative basis for estimating the likely range of the next follow‐up measurement, identifying deviations from expected change, and optimizing longitudinal‐monitoring research.

This study also extends the current scope of machine learning in liver disease. Fibro‐Predict used nationwide electronic health records to identify the risk of advanced fibrosis and underwent temporal validation and prospective clinical validation [28]. The hepatocellular‐carcinoma risk model SMART‐HCC combined LSM with clinical variables and was evaluated in independent Asian and European cohorts [29]. In contrast to these clinical‐event or classification models, our study focuses on future continuous LSM and reports MAE, RMSE, *R* ^2^, continuous‐outcome calibration, and direct comparisons with several simple longitudinal strategies. This design provides evidence for continuous‐outcome prediction in routine follow‐up data that complements existing risk‐classification models.

The measurement characteristics of transient elastography further demonstrate the relevance of continuous prediction. The technology was originally introduced as a reproducible method for measuring liver stiffness [30], and an early prospective study compared it with liver biopsy, FibroTest, and the aspartate aminotransferase‐to‐platelet ratio index (APRI), demonstrating its diagnostic performance for assessing hepatitis C–related fibrosis [31]. At the same time, LSM reflects dynamic physiological and pathological information related to food intake [32], acute inflammation, and aminotransferase changes [33]. Stiffness thresholds for fibrosis stages vary widely and overlap across studies [34], and a disease‐specific dual‐cutoff strategy has been externally validated in primary biliary cholangitis [35]. By directly modeling continuous LSM, our study is aligned with the focus on dynamic stiffness changes in longitudinal monitoring. Calibration point estimates remained close to ideal despite real‐world measurement heterogeneity, suggesting that routine follow‐up records contain a stable longitudinal signal that can be learned by the model.

Longitudinal outcome studies further broaden the potential applications of this model. Long‐term natural‐history data indicate that clinically meaningful liver events generally occur over timescales longer than the approximately 12‐month prediction window used in this study [36]. Serial FIB‐4 trajectories are associated with subsequent risks of cirrhosis and hepatocellular carcinoma [37], and multimodal research has compared multiparametric magnetic resonance imaging, transient elastography, and blood fibrosis markers for predicting clinical outcomes within the same cohort [38]. A large registry study has also reported associations between stiffness trajectories and the risk of liver‐related events [39]. The continuous‐LSM prediction framework established here may therefore provide a methodological bridge between short‐term measurement changes and research on long‐term clinical outcomes.

The performance of the simple benchmarks also provides valuable clinical context for the model results. The low errors of the last observed LSM and historical mean reflect the persistence of liver stiffness between adjacent follow‐up visits. LSM represents both fibrosis burden and dynamic information related to inflammation, cholestasis, food intake, exercise, and venous congestion [14], while its longitudinal changes may also reflect treatment response and biochemical activity [25]. The random forest integrated current measurement status, historical distribution, follow‐up timing, and routine clinical indicators within a unified nonlinear framework, reducing the MAE of linear‐trend extrapolation from 12.066 kPa to 3.622 kPa in the 2023 test set, an error reduction of approximately 70%. The locked multivariable framework retained its advantage over linear‐trend extrapolation in real‐world follow‐up with few historical measurements and uneven intervals, while providing a unified basis for individualized extensions incorporating treatment, etiology, and disease activity.

Feature‐level results showed that hist_median, hist_mean, and last_lsm retained high permutation importance in both temporal holdout cohorts, indicating that the locked model primarily used prior LSM level and its historical distribution. Because these summaries are correlated, permutation importance reflects the locked model’s predictive reliance rather than an independent clinical or causal effect of any single variable. The small performance changes after removing one feature in LOFO are consistent with information sharing and compensation among correlated longitudinal features. This analysis identifies the model’s core information layer and prioritizes candidate variables for subsequent incremental modeling of standardized clinical and multimodal information.

Point estimates of the global calibration slopes were close to 1 and the intercepts were close to 0 in both temporal holdout cohorts, suggesting no marked overall linear calibration shift in the later cohorts; CIs for the calibration parameters were wide, with detailed uncertainty reported in the Results and Figure 3. In the 90‐day complete‐case subset, the random forest had a lower MAE point estimate than the FIB‐4 linear mapping, but this comparison provides only an exploratory, task‐aligned reference. The last observed LSM remained the transparent benchmark in the same subset, and the FIB‐4 comparison should not be interpreted as evidence of clinical superiority.

The established noninvasive‐test literature also highlights the complementary value of the longitudinal prediction perspective used here. APRI was originally developed from routine aminotransferase and platelet measurements for fibrosis assessment [40]; FIB‐4 was subsequently validated as a low‐cost marker in hepatitis C [41]; and the nonalcoholic fatty liver disease fibrosis score was developed in biopsy‐confirmed nonalcoholic fatty liver disease to identify advanced disease cross‐sectionally [42]. Repeated FIB‐4 measurements can also help identify individuals at risk of severe liver disease [43], and relevant clinical guidelines have incorporated serum scores and imaging‐based noninvasive tests into staged diagnostic and risk‐assessment pathways [44–47]. While these tools primarily assess current fibrosis, our study focuses on continuous LSM at the next time point and thus adds a dynamic predictive dimension to noninvasive liver‐disease assessment.

Biomarker research provides a clear route toward further improvement in model performance. Collagen‐turnover markers may reflect extracellular‐matrix biology that cannot be fully captured by a single mechanical stiffness measurement [48]. Proteomic or multianalyte panels have shown potential for the noninvasive assessment of fibrosis in metabolic dysfunction–associated steatotic liver disease [49], and a recent review also emphasized the importance of analytical standardization, clinical validation, and clearly defined boundaries of use [50]. Combining standardized biomarkers, etiology, treatment exposure, and disease‐activity indicators with the existing longitudinal LSM features could further improve individualized prediction within the temporal forecasting framework established in this study.

Several established frameworks also support our evaluation strategy. The Prediction model Risk Of Bias ASsessment Tool (PROBAST) organizes prediction‐model assessment across participants, predictors, outcomes, and analysis [51], while general performance‐evaluation frameworks emphasize distinguishing prediction error, calibration, and discrimination and matching metrics to the intended use [52]. Research on calibration stability [53] and sample size for the development and external validation of continuous‐outcome models [54, 55] further underscores the importance of evaluating calibration, uncertainty, and sample adequacy. In addition, a systematic comparison of machine learning with logistic regression found no consistent performance advantage for machine learning and identified continuing methodological and reporting shortcomings in model comparisons [56]. In accordance with these principles, we evaluated three commonly used algorithms using nested temporal validation and fixed the optimal pipeline before applying it to later cohorts, thereby improving the transparency and reproducibility of performance estimates.

This study has three principal limitations. First, it is a retrospective analysis from a single‐institution data source and includes only patients who remained under follow‐up and had a subsequent LSM, creating potential selection bias; its applicability across institutions and different clinical populations still requires multicenter external validation. Second, the test and validation sets included only 178 and 103 patients, respectively, and some subgroups were small, limiting the precision of the corresponding results, which should be regarded as exploratory estimates. Third, this study predicts continuous LSM approximately 12 months later rather than liver histology or patient‐important outcomes such as decompensation, hepatocellular carcinoma, transplantation, or death; routine records also incompletely capture acquisition conditions, etiology, treatment exposure, inflammatory activity, and comorbidities, limiting clinical interpretation of the model and assessment of incremental value.

Overall, this study demonstrates the feasibility of predicting continuous LSM approximately 12 months ahead using routine longitudinal transient elastography and clinical indicators and establishes a quantitative framework with temporal reproducibility, overall calibration, and feature‐level interpretability. Future studies can build on this framework by standardizing transient‐elastography acquisition, adding information on etiology, treatment changes, inflammatory activity, comorbidities, and novel biomarkers, and conducting independent multicenter validation, thereby extending continuous‐LSM prediction into a tool for individualized follow‐up and dynamic monitoring.

## 5. Conclusions

A random forest based on longitudinal transient elastography and routine clinical indicators achieved out‐of‐time prediction of continuous LSM approximately 12 months ahead in the 2023 test set and 2024 validation set, with MAEs of 3.622 and 2.989 kPa, *R* ^2^ values of 0.456 and 0.432, and calibration slopes of 1.043 and 0.951, respectively; relative to linear‐trend extrapolation, it reduced MAE by 8.443 kPa in the 2023 test set. hist_median, hist_mean, and last_lsm consistently ranked among the most important features in both temporal holdout cohorts, indicating that the model extracted a core signal from longitudinal LSM history that remained consistent in later temporal cohorts. The rigorous patient‐level temporal split, nested validation, locked pipeline, and comparison with transparent benchmarks provide a reproducible methodological basis for continuous‐LSM prediction, dynamic follow‐up, and subsequent multicenter extension.

## Author Contributions

Ming Lei: investigation, resources, methodology, software, validation, project administration, and visualization. Shoukang Zou: investigation, methodology, validation, and visualization. Qing Liu: conceptualization, project administration, and supervision. Yangling Li: data curation, formal analysis, validation, and investigation. Jing Han: resources, software, validation, and investigation. Yungang Zhang: formal analysis and project administration. Chunwen Pu: conceptualization, funding acquisition, formal analysis, project administration, and writing–original draft.

## Funding

This work was supported by the Dalian Municipal Bureau of Science and Technology through the following projects: “A nested case‐control study within a large longitudinal cohort to support the prevention and treatment of hepatitis B‐related liver cancer throughout the disease course” (Grant No. 2024JJ13PT062) and “Clinical utility and underlying mechanisms of chitinase‐3‐like protein 1 as a non‐invasive biomarker for assessing liver fibrosis” (Grant No. 2023‐243‐2).

## Ethics Statement

This retrospective observational study was conducted in compliance with the Declaration of Helsinki and its subsequent amendments, as well as relevant national and institutional ethical guidelines. The study protocol was reviewed and approved by the Institutional Review Board of Dalian Public Health Clinical Center (Approval No.: 2024‐029 (KY) ‐002). All patients included in the study provided written informed consent prior to participation, authorizing the collection, use, and retrospective analysis of their clinical data (including longitudinal liver stiffness measurement results, biochemical indicators, and follow‐up information) for research purposes. For patients who were unable to provide consent independently, informed consent was obtained from their legally authorized representatives. Strict measures were implemented to protect patient privacy and data security throughout the study process. All personal identifiable information (e.g., name, medical record number, and contact information) was de‐identified and anonymized prior to data analysis. The dataset was stored in a password‐protected secure server with restricted access, and only authorized researchers were permitted to use the data for the approved research objectives. No personal information was disclosed or shared with third parties.

## Conflicts of Interest

The authors declare no conflicts of interest.

## Supporting Information

Additional supporting information can be found online in the Supporting Information section.

## Supporting information


**Supporting Information 1** Supporting Table 1. Details of nested expanding temporal cross‐validation, including fold dates, sample sizes, selected parameters, and training and validation performance.

**Supporting Information 2** Supporting Table 2. Nonexclusive diagnosis‐text evidence in prior records for hepatitis B, hepatitis C, alcohol‐related liver disease, fatty liver, and autoimmune or cholestatic disease, shown for the train set, test set, validation set, and overall cohort. Values are *n* (%), and categories may overlap; these indicators were used for cohort description and not as model predictors.

**Supporting Information 3** Supporting Figure 1. Exploratory clustering diagnostics. (A) Silhouette coefficients for *k* = 2–6 in the train set. (B) Distribution of bootstrap estimates of the adjusted Rand index at *k* = 2.

## Data Availability

The analysis code supporting the findings of this study is openly available at https://github.com/sunmichace/20250812_Liu_LiverHarderML2. The deidentified patient‐level data are not publicly available because of ethical and privacy restrictions but are available from the corresponding authors upon reasonable request.
